# Nanoencapsulation of Thyme Essential Oils: Formulation, Characterization, Storage Stability, and Biological Activity

**DOI:** 10.3390/foods11131858

**Published:** 2022-06-23

**Authors:** Asma Jayari, Francesco Donsì, Giovanna Ferrari, Abderrazak Maaroufi

**Affiliations:** 1 Group of Bacteriology and Biotechnology Development, Laboratory of Epidemiology and Veterinary Microbiology, Institute Pasteur of Tunis, BP 74, 13 Place Pasteur, Belvédère, Tunis 1002, Tunisia; asma_jayari@hotmail.fr (A.J.); abderrazak.maaroufi@pasteur.tn (A.M.); 2 Department of Industrial Engineering, University of Salerno, Via Giovanni Paolo II 132, 84084 Fisciano, Italy; gferrari@unisa.it; 3 ProdAl Scarl, University of Salerno, Via Giovanni Paolo II 132, 84084 Fisciano, Italy

**Keywords:** essential oils, *Thymus capitatus*, *Thymus algeriensis*, nanoemulsions, nanoparticles, physical stability, antioxidant activity, antibacterial activity

## Abstract

This study aimed to improve the effectiveness of *Thymus capitatus* and *Thymus algeriensis* essential oils (EOs), as food preservatives, through their encapsulation in different delivery systems (DSs), namely nanoemulsions and biopolymeric nanoparticles. DSs’ preparation is tailored to enhance not only physical stability but also resulting Eos’ antioxidant and antibacterial activities through different fabrication methods (high-pressure homogenization emulsification or antisolvent precipitation) and using different emulsifiers and stabilizers. DSs are characterized in terms of droplet size distribution, ζ-potential, and stability over time, as well as antioxidant and antibacterial activities of encapsulated EOs. The antioxidant activity was studied by the FRAP assay; the antibacterial activity was evaluated by the well diffusion method. EOs of different compositions were tested, namely two EOs extracted from *Thymus capitatus,* harvested from Tunisia during different periods of the year (TC1 and TC2), and one EO extracted from *Thymus algeriensis* (TA). The composition of TC1 was significantly richer in carvacrol than TC2 and TA. The most stable formulation was the zein-based nanoparticles prepared with TC1 and stabilized with maltodextrins, which exhibit droplet size, polydispersity index, ζ-potential, and encapsulation efficiency of 74.7 nm, 0.14, 38.7 mV, and 99.66%, respectively. This formulation led also to an improvement in the resulting antioxidant (60.69 µg/mg vs. 57.67 µg/mg for non-encapsulated TC1) and antibacterial (inhibition diameters varying between 12 and 33 mm vs. a range between 12 and 28 mm for non-encapsulated TC1) activities of EO. This formulation offers a promising option for the effective use of natural antibacterial bioactive molecules in the food industry against pathogenic and spoilage bacteria.

## 1. Introduction

In recent years, the use of synthetic preservatives has been progressively reduced in the food industry because some of them are reported to exhibit toxic effects, which could be, in the long term, carcinogenic [1,2]. Therefore, consumers tend to prefer healthier foods, prepared with natural or naturally derived ingredients, and with cleaner labels. This has prompted scientists to investigate natural products that possess antimicrobial and antioxidant activities as alternatives to chemical preservatives. Aromatic plants have been traditionally used for centuries for seasoning and extending the shelf-life of food [3]. Their antimicrobial properties are due to their content in essential oils (EOs), formed through their secondary metabolism [4]. Among available EOs, many are characterized by sufficiently high antioxidant, antibacterial and antifungal activities, making them suitable for use as natural preservatives [5]. However, EOs are only scarcely soluble in water, hence reducing their effectiveness as antimicrobial agents when added to food [6].

Moreover, many EOs are not as effective as commonly used synthetic additives or may interact with food components, such as phenolic compounds, proteins, or fats, decreasing the resulting antimicrobial effects [7,8]. Consequently, generally high concentrations of EO are required for effective antimicrobial action to ensure the desired product shelf-life. Because of the intense aroma of EOs, this could lead to alteration of the product’s sensory attributes, further limiting their application in food [9,10,11].

In fact, in meats, the use of EOs contributed to pathogen control, hence reducing the risk of foodborne outbreaks and ensuring safe meat products for the consumers [11,12]. However, the hydrophobic nature of EOs and their high reactivity with product matrix represent a huge challenge for their direct incorporation into food products [13].

To overcome these shortcomings, different delivery systems (DSs) have been developed, allowing the incorporation of essential oils in hydrophilic carriers for their efficient dispersion into food products [14], ensuring natural formulation, ease of production, and compatibility with the food products [6].

In this perspective, encapsulation provides effective protection of antimicrobial compounds against chemical reactions and undesirable interactions with other food components; it would improve their solubility, decrease their migration, and preserve their bioactive stability during processing and storage [15]. Additionally, encapsulation is reported to contribute also to controlling the release of encapsulated compounds, as well as their bioaccessibility and bioavailability [6]. While micro-encapsulation systems can guarantee the protection of antimicrobial compounds against degradation or evaporation, the high surface-to-volume ratio of nano-encapsulation systems (nanometer-scale systems, below 100 nm) can increase the concentration of antimicrobials in specific food areas where microorganisms would preferentially proliferate and improve absorption mechanisms of passive cells that may enhance antimicrobial activities [16,17].

Among the nanoencapsulation systems currently used for the delivery of bioactive compounds, nanoemulsions have been proven suitable for use in food products due to their ease of preparation and desirable functional attributes [16,18,19]. The small droplet sizes can increase the interactions between active compounds and biological membranes, as well as promote their transfer across them. Additionally, nanoemulsions can be designed with good kinetic stability and low turbidity for a wide range of commercial applications [20], as preservatives for food, beverages, cosmetics, and pharmaceuticals [21,22]. Various low- and high-energy methods have been applied to produce nano-emulsions [23]. However, nanoemulsion production using natural emulsifiers is typically based on high-energy methods, such as high-pressure homogenization [24].

Biopolymeric nanoparticles also represent a class of DSs, which is specifically suitable for food application, because of the high compatibility of the matrix materials (macromolecules, such as proteins or polysaccharides) with food products [6]. Zein, the storage protein in corn, is a biopolymer especially appropriate for preparing nanoparticles, due to its biocompatibility, biodegradability, non-toxicity, hydrophobicity, and solubility in concentrated ethanol solutions [25], which make it suitable for encapsulating both hydrophobic and hydrophilic bioactive compounds [26,27].

In the present work, the main goal was to prepare stable nanometric DSs with food-grade materials (e.g., proteins, polysaccharides, and oils), for encapsulation of EOs as antimicrobial agents. In particular, thyme EOs were tested in non-encapsulated and encapsulated forms, comparing their antioxidant and antibacterial activity against five food-borne pathogens (*Escherichia coli, Listeria monocytogenes, Pseudomonas aeruginosa, Salmonella typhimurium,* and *Staphylococcus aureus*).

The purposes of this study were (i) to determine the effect of emulsifier type and concentration on nanoemulsion droplet size distribution, (ii) the effect of the type of stabilizer on nanoparticle properties, and (iii) the effect of the essential oil composition on the delivery systems characteristics.

## 2. Materials and Methods

### 2.1. Materials and Chemicals

The tested EOs were extracted from *Thymus capitatus* (harvested in July 2018) (TC1), *Thymus capitatus* (harvested in April 2018) (TC2), and *Thymus algeriensis* (harvested in April 2018) (TA), collected from the north of Tunisia (Zaghouan); latitude 36.4028″ (N); longitude 10.1433″ (E) and altitude of 176 m.

The following materials were used for the fabrication of the DSs: sunflower oil (SO) was bought from a local market (Olio di Semi di Girasole Basso, San Michele di Serino (AV), Italy), Tween 80 (Polysorbate 80, CAS no. 9005-65-6) (T80), pectin (from apple, CAS no. 9000-69-5) (PEC), Quillaja saponin (sapogenin content 20–35% wt., CAS no. 8047-15-2) (QS), gum arabic (from Acacia tree, CAS no. 9000-01-5) (GA), maltodextrin (dextrose equivalent of 16.5–19.5, CAS no. 9050-36-6) (MD) and zein (CAS no. 9010-66-6) (ZN) were bought from Sigma-Aldrich S.r.l. (Milan, Italy), soy lecithin (Soy lecithin Solec IP) (LEC) was obtained from Solae Italia S.r.l. (Milan, Italy), and whey proteins (WP) (see composition in [28]), with an average molecular weight of 18.2 kDa (Volactive UltraWhey 90), were obtained from Volac Socoor S.r.l. (Milan, Italy). All other chemicals and solvents used in this study were purchased from Sigma Aldrich S.r.l. (Milan, Italy), unless otherwise specified.

### 2.2. Methods

#### 2.2.1. Determination of Composition and Carvacrol Concentration of Essential Oils

Analyses of essential oils were carried out using a Thermo-Finnigan GC-MS, equipped with an Agilent DB-5 MS capillary column (30 m × 0.250 mm). For GC/MS detection, the ion source was set to 250 °C, Inlet: 230 °C, Line X: 250 °C, Oven: 60 °C for 3 min, then at a rate of 3 °C/min at 100 °C and held for 1 min, then at a rate of 5 °C/min at 140 °C and maintained for 1 min and finally at a rate of 20 °C/min at 240 °C and maintained for 5 min. The carrier gas was helium at a flow rate of 1.0 mL/min. 500 µL of EO was diluted in 4 mL of dichloromethane before manual injection (1 µL). The identification of the oil components was based on their retention times, compared to those of a homologous series of n-alkanes, and comparison of their mass spectral fragmentation patterns with those reported in the NIST Mass Spectral Library as well as an in-house library. Carvacrol concentration in the different EOs was quantified through comparison with a carvacrol standard (98% purity, CAS: 499-75-2, Sigma-Aldrich S.r.l., Milan, Italy).

#### 2.2.2. Fabrication of the Delivery Systems

The DSs were prepared with different approaches, according to the formulations and methods presented in Table 1. In the case of nanoemulsions stabilized with T80, LEC, LEC/PEC, WP, and QS, primary emulsions were prepared by adding dropwise the oil phase to a buffered aqueous phase solution (phosphate buffer at pH = 7.4 [29]) containing the emulsifier at the desired concentration (see Table 1), to reach a total volume of 200 mL, under stirring using an Ultra Turrax T25 mixer (IKA Werke GmbH & Co. KG, Staufen im, Breisgau, Germany) at 20,000 rpm for 5 min, in an ice bath, to prevent the degradation of the EOs. Subsequently, the nanoemulsions were produced by high-pressure homogenization (HPH) of the primary emulsions, using a homogenizer developed in-house, equipped with a 100 μm orifice valve (model WS1973, Maximator JET GmbH, Schweinfurt, Germany), operated through an air-driven Haskel pump model DXHF-683 (EGAR S.r.l., Milan, Italy) [28]. The HPH treatment consisted of five passes at 100 MPa, with an intermediate cooling at 5 °C in a tube-in-tube heat exchanger.

The zein-based nanoparticles were prepared using the antisolvent precipitation method, according to the protocol described by Gali et al. [27], with some modifications. Briefly, 1% wt. ZN and 0.4% wt. EO were dissolved in an aqueous solution of 80% wt. ethanol. Solutions of PEC, GA, and MD were separately prepared in distilled water at a concentration of 0.33% wt. under magnetic stirring for 5 h and stored overnight at room temperature. An amount of 25 g of the zein solution, previously prepared, was added dropwise to 75 g of each PEC, GA, and MD stabilizer solution with continuous stirring (400 rpm) for 30 min. Zein particles are also precipitated in pure water (without a stabilizer) to better highlight the contribution of the stabilizers. The samples were subjected to ethanol removal under reduced pressure at 30 °C, using a rotary evaporator (Rotavapor R-114). The removed volume (about 75% of the initial volume) was replaced with distilled water. The different suspensions were adjusted at pH 4 and stored at 4 °C.

#### 2.2.3. Mean Droplet Size, Polydispersity Index, and ζ-Potential Measurements

The mean droplet size (hydrodynamic diameter, d_H_), polydispersity index (PDI), and ζ-potential of the different DSs were measured using a Zetasizer Nano (Malvern Instruments Ltd., Malvern, UK), by a dynamic light scattering measurement of Brownian motions or electrophoretic motion of suspended particles or droplets at 25 °C. Each measurement was the average of 3 trials [28]. For the ζ-potential measurements, dilutions with a phosphate-buffered solution at pH = 7.4 were necessary to obtain better quality measurements. The stability of the DSs was evaluated at 25 °C by measuring d_H_ and ζ-potential changes over time.

#### 2.2.4. Encapsulation Efficiency Determination

The encapsulation efficiency of the different formulations was evaluated according to a procedure based on previously proposed methods [30]. Briefly, the bulk EOs in the aqueous phase ($m_{EO}^{Aq}$) was quantified and deducted from the total amount of encapsulated EOs ($m_{EO}^{Tot}$). For this purpose, 1 mL of the sample and 2 mL of pure ethanol were homogenized by vortexing for 1 min. Then, the solution was centrifuged at 14,000 rpm (16,000× *g*) for 5 min (Micro Centrifuge 5415C, Eppendorf S.r.l., Milan, Italy). One mL of supernatant and 5 mL of n-hexane were homogenized by vortexing for 1 min. The supernatant was removed, and sodium sulfate was added to the pellet. The resulting mixture was filtered through 0.2 µm PTFE filters (Acrodisc^®^, PALL Italia S.r.l., Milan, Italy). The absorbance of the blue color was read at 745 nm using a UV-Visible spectrophotometer (Jasco V-650, JASCO Europe S.r.l., Lecco (MI), Italy). A standard calibration curve was obtained for different EOs concentrations. The encapsulation efficiency (EE%) was calculated using Equation (1) as follows:

(1) $$EE\left(\%\right)=\frac{m_{EO}^{Tot}-m_{EO}^{Aq}}{m_{EO}^{Tot}}$$

#### 2.2.5. Antioxidant Activity Measurements

The antioxidant activity of DSs was assessed using the ferric reducing antioxidant power (FRAP) assay, as previously described by Guo and Jauregi [31], with some modifications. FRAP reagent was freshly prepared by mixing at a ratio of 10:1:1 acetate buffer, 300 mM (pH 3.6, consisting of 3.1 g sodium acetate and 16 mL acetic acid icy dissolved in 1 L of distilled water), 20 mM ferric chloride hexahydrate (FeCl_3_·6H_2_O) and 10 mM 2,4,6-tripyridyl-s-triazine (TPTZ) in 40 mM hydrochloric acid (HCl). An amount of 2.5 mL of reagent was mixed with 500 μL of the sample and incubated at room temperature for 10 min. The absorbance was then read at 593 nm in a UV-Visible spectrophotometer (Jasco V-650 spectrophotometer). An ascorbic acid solution was used to obtain a calibration curve and the results were expressed in µg of ascorbic acid equivalent (AAE)/mg of extract; each measurement being reproduced three times.

#### 2.2.6. Antibacterial Activity Tests

The inhibitory effect of the EOs and DSs was tested against five pathogenic bacteria by diffusion in wells, according to the protocol described by Cintas et al. [32] with some modifications. *Escherichia coli* ATCC 25922, *Pseudomonas aeruginosa* ATCC 27853, *Salmonella typhimurium* ATCC 14028, *Staphylococcus aureus* ATCC 25923, and *Listeria monocytogenes* ATCC 19,118 were inoculated onto nutrient agar media and incubated for 24 h. From these young cultures and using a platinum loop, a few well-isolated and identical colonies were selected and placed in 5 mL of Brain Heart Infusion (BHI) broth. The bacterial suspension was well homogenized and incubated for 18 h. Then, 1 mL of each test culture was deeply inoculated with approximately 10^6^ UFC/mL in agar. After the medium solidified, wells of 6 mm in diameter were prepared under sterile conditions. A volume of 100 µL of selected DSs at an EO concentration of 5% wt. was added to each well. The dishes were pre-incubated at 4 °C for 2 h for total diffusion of DSs followed by incubation at 37 °C for 24 h. The antibacterial activity was evaluated by measuring the diameter of the inhibition zones (mm) around the wells. The experiment was carried out in triplicate.

#### 2.2.7. Statistical Analysis

Data were reported as means ± standard deviation of three measurements. The analysis of variance was performed by a one-way ANOVA analysis using Graph Pad Prism 5.0 software (Dotmatics, San Diego, CA, USA), followed by Tukey’s multiple comparison test. Means are considered significantly different at *p* < 0.05.

Data related to particle size, PDI, and ζ-potential were analyzed by one-way ANOVA with the general linear model procedure of SAS (version 9.1, SAS, Cary, NC, USA). The residual mean square error was used as the error term. Means were separated using the Duncan test with a significance level of *p* < 0.05 (SAS, 9.1).

## 3. Results

### 3.1. Composition of Thyme Essential Oils

The results of the GC-MS analysis of *Thymus capitatus* EO revealed the presence of 13 different compounds belonging to the phenolic and terpenic groups (Table 2). Similar results have been reported by Hassan et al. [33]. Moreover, previous studies have indicated that the major component of thyme oil is carvacrol [34]. Conversely, GC-MS analysis of EO from *Thymus algeriensis* revealed the identification of 21 bioactive compounds. For the three EOs considered, the majority of their compounds were phenols (carvacrol and thymol), followed by terpenes. Indeed, Gonçalves et al. reported that carvacrol is the major constituent of *Thymus capitatus* EO (TC) [35]. On the other hand, the major compounds of the EO of *T. algeriensis* (TA), as identified by Nikolic et al. [36], are thymol and carvacrol. However, Dob et al. [37] reported that the EO of TA is dominated by linalool and thymol. The EOs of TC1, TC2, and TA showed differences in their carvacrol concentrations (determined through comparison with a carvacrol standard) with a predominance in TC1 (907 g/L), which was harvested in July. These results, with a strong predominance of carvacrol, are well aligned to previous results for EO from TC harvested in June [34]. As for TC2 and TA, the carvacrol concentrations were around 50.9 and 63.6 g/L, respectively. Our results suggested that variation in their chemical compositions could be due to the variability of the species studied and the harvesting period of the plant.

### 3.2. Mean Size and Polydispersity Index

**Figure 1:**
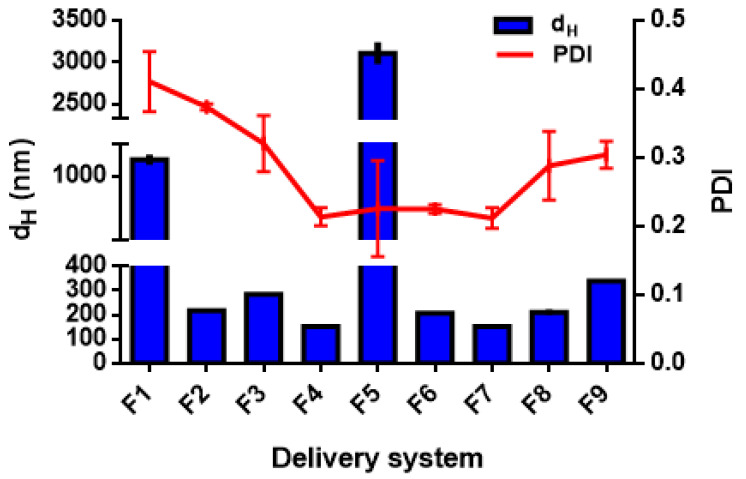
Hydrodynamic diameter (d_H_) and polydispersity index (PDI) of TC1 EO nano-emulsions (formulations F1–F9 in Table 1).

**Figure 2:**
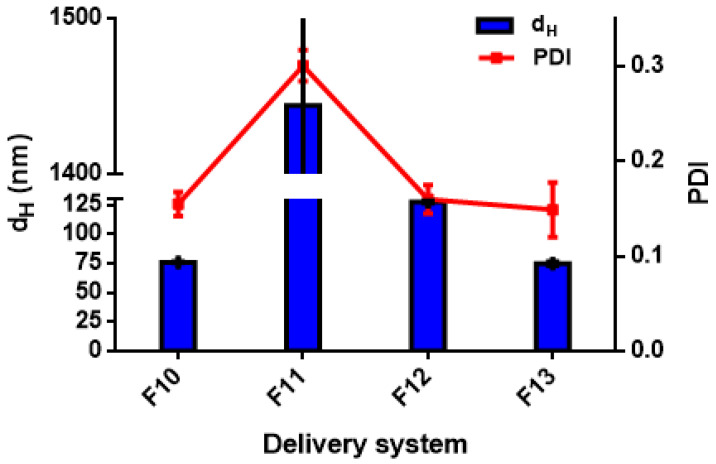
Hydrodynamic diameter (d_H_) and polydispersity index (PDI) of TC1 EO nanoparticles (formulations F10–F13 in Table 1).

**Figure 3:**
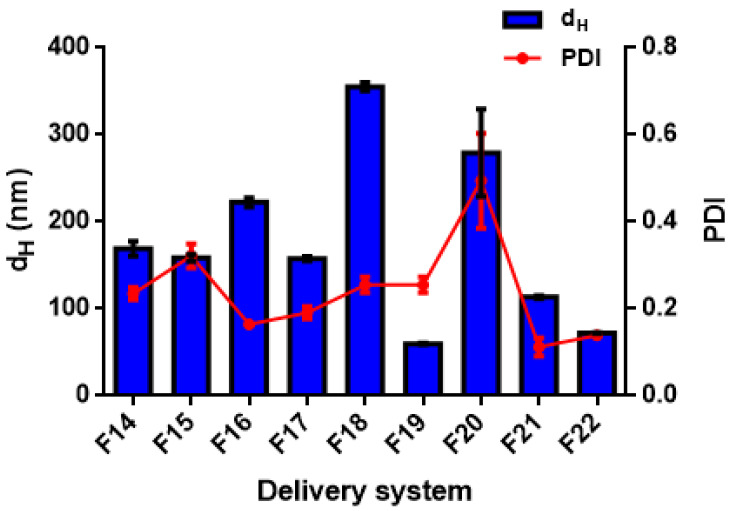
Hydrodynamic diameter (d_H_) and polydispersity index (PDI) of TC2 EO nanoemulsions (formulations F14–F18 in Table 1) and nanoparticles (formulations F19–F22 in Table 1).

**Figure 4:**
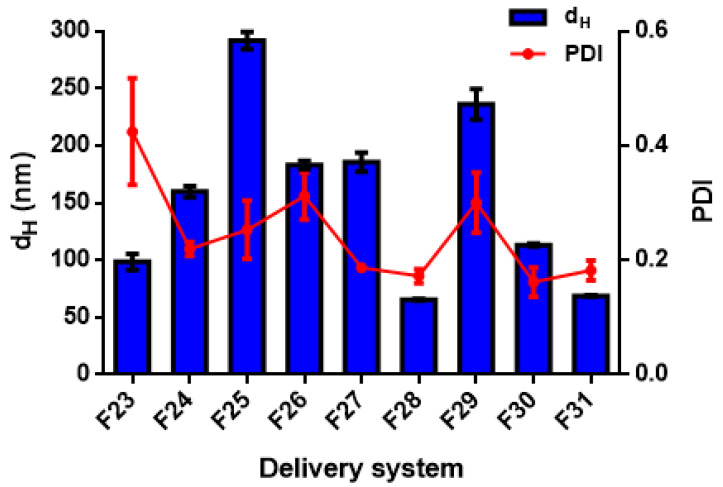
Hydrodynamic diameter (d_H_) and polydispersity index (PDI) of TA EO nanoemulsions (formulations F23–F27 in Table 1) and nanoparticles (formulations F28–F31 in Table 1).

**Figure 5:**
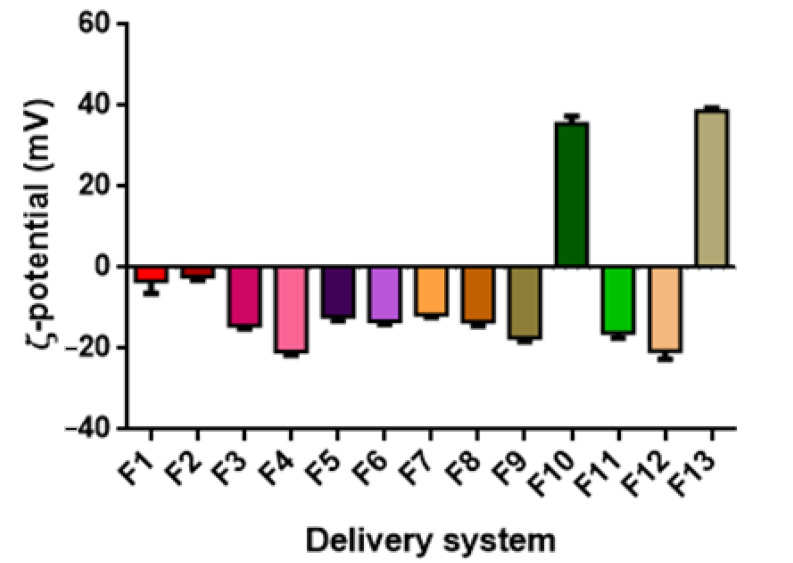
ζ-potential of TC1 EO nanoemulsions (formulations F1–F9) and nanoparticles (formulations F10–F13).

**Figure 6:**
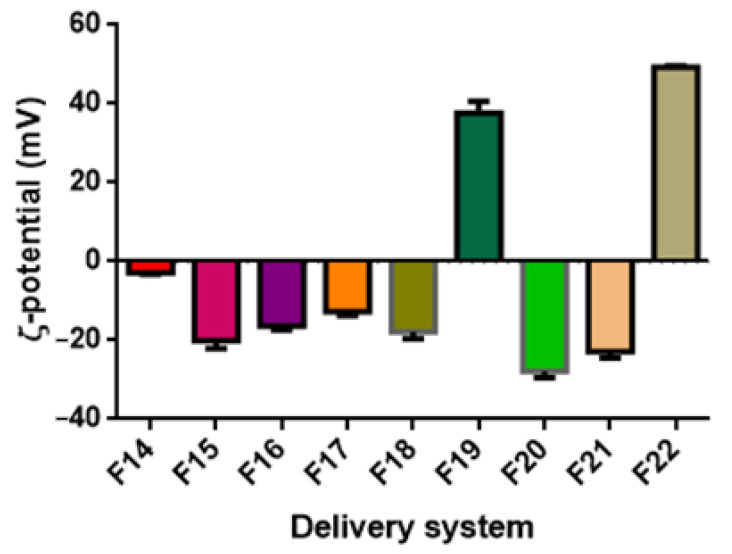
ζ-potential of TC2 EO nanoemulsions (formulations F14–F18) and nanoparticles (formulations F19–F22).

**Figure 7:**
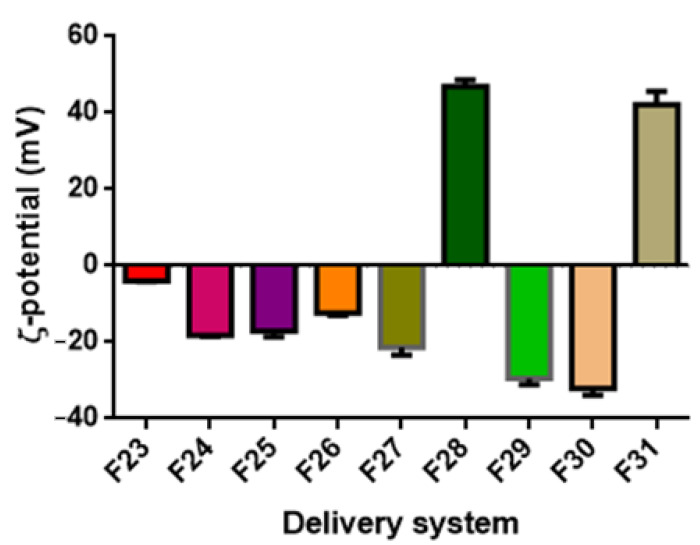
ζ-potential of TA EO nanoemulsions (formulations F23–F27) and nanoparticles (formulations F28–F31).

Different formulations were tested to manufacture the DSs, as reported in Table 1, using either high- (for nanoemulsions) or low-energy methods (for biopolymeric nanoparticles) [38]. In this work, the different formulations were fabricated using a high-pressure homogenization method and an anti-solvent precipitation technique. The mean droplet hydrodynamic diameters (d_H_) and the polydispersity indexes are generally used for a preliminary assessment of the DSs; it is generally accepted that the smaller the size of the droplets or particles, the narrower the size distribution (PDI < 0.3), the more likely the DSs are stable, and the more favourable the bio-accessibility of the payload compounds [39]. Mean size (d_H_) and polydispersity index (PDI) are reported for all the data in Table 3 and shown for detailed discussion in Figure 1, Figure 2, Figure 3, Figure 4, Figure 5, Figure 6 and Figure 7.

#### 3.2.1. Effect of Emulsifier Type and Concentration on Nanoemulsion Droplet Size Distribution

The DS formulations F1–F9 correspond to nanoemulsions prepared via HPH. Their d_H_ and PDI are shown in Figure 1. For the F1 and F2 formulations, based on T80, the finest size was obtained for the formulation F2, containing 1.5% wt. T80 and 1.5% wt. TC1 EO (emulsifier/oil phase ratio = 1:1 *w*/*w*), with d_H_ = 218.4 ± 2.4 nm and PDI = 0.37± 0.03. In contrast, formulation F1, containing 0.5% wt. T80 and 1% wt. TC1 EO (emulsifier/oil phase ratio = 1:2 *w*/*w*) presented significantly higher size (d_H_ = 1099.0 ± 33.0 nm) and polydispersity (PDI = 0.41± 0.04). In the case of lecithin-based formulations (F3, F4), the F3, characterized by an emulsifier/oil phase ratio = 1:1 *w*/*w* (containing 1% wt. LEC and 1% wt. TC1 EO), exhibited a d_H_ = 284.6 ± 3.8 nm and a PDI of 0.32 ± 0.04. Increasing the concentration of LEC with respect to the oil phase (formulation F4, with an emulsifier/oil phase = 2:1 *w*/*w*), induced a significant (*p* < 0.0001) reduction in the size, with d_H_ = 152.8 ± 2.2 nm and PDI = 0.21 ± 0.01. Ozturk et al. [40] reported that a concentration of 2% wt. lecithin is required to form small droplets with a diameter <150 nm, for an oil phase of 10% (*w*/*w*). Donsi and Ferrari [41] have used lecithin (emulsifier/oily phase ratio of 1:2 *w*/*w*) to encapsulate carvacrol by HPH (3 passes and 200 MPa) and obtained d_H_ = 179.7 ± 2.9 nm and PDI = 0.22 ± 0.02.

PEC is a polysaccharide, mainly used for its gelling and stabilizing properties as well as its capacity of preventing coalescence of the formed droplets. In particular, PEC is reported to stabilize fine emulsions by electrostatic repulsion and steric effect [42]. However, an excess of polymer concentration might lead to bridging flocculation [24]. The incorporation of PEC as a stabilizer into LEC-based formulations (F5 and F6) caused an increase in the droplet size. However, addition of PEC in formulation F5 (0.66% wt. PEC) caused emulsion destabilization, with d_H_ = 3108.0 ± 132.6 nm and PDI = 0.22 ± 0.06. In contrast, for F6, only a slight size increase was observed (with respect to F4), because of lower PEC concentration (0.16% wt.), with d_H_ = 206.6 ± 1.7 nm and PDI = 0.22 ± 0.01.

Whey proteins can also be used as an emulsifier, as they feature both hydrophilic and lipophilic residues and high surface activity, with the capability to form cohesive and strong films around the oil droplets [43,44,45]. The nanoemulsion based on WP, with an emulsifier/EO ratio = 2:1 *w*/*w* (F7), resulted in d_H_ = 210.8 ± 11.7 nm and PDI = 0.28 ± 0.05. In addition, the formulation F8, containing 0.1% wt. EO, 0.1% wt. sunflower oil and 0.4% wt. WP (emulsifier/oil phase ratio = 2:1), resulted in finer droplets, with d_H_ = 152.2 ± 1.3 nm and PDI = 0.21 ± 0.01. These results are similar to those found by Tastan et al. [46], who showed that WP-based carvacrol nanoemulsions can be produced with d_H_ = 115.0 ± 10.0 nm and PDI = 0.24 ± 0.04. Indeed, the ability of WP to form nanometric droplets may be related to the rapid adsorption of proteins onto the droplet surface, forming thin but compact interfacial layers [47].

The results shown in Figure 1 suggest that the emulsion droplet size strongly depends on the concentration of the emulsifier. Droplet size decreased considerably with the increase in the concentration of the emulsifier, as confirmed by Qian and Mc Clements [48] because more emulsifier is available to cover the surface of the droplets formed during the homogenization process [49,50,51], and faster coverage of the droplet surface occurs, resulting in lower interfacial tension [50]. Moreover, by increasing the concentration of the emulsifier, the PDI also decreases, indicating that the formed droplets tend to be uniformly and tightly distributed [43].

However, from a practical point of view, it is generally advantageous to use the minimum amount of emulsifier necessary to form stable emulsions, as this would reduce cost, undesirable taste, and toxicity. Additionally, high concentrations of non-adsorbed emulsifiers can decrease emulsion stability, promoting Ostwald ripening or droplet flocculation [52,53]. It is also important to note that increasing the percentage of EO would lead to the formation of larger droplets and the instability of the emulsion. Such a result was confirmed by the work of Mazarei and Rafati [54], showing that the effect of the emulsifier’s concentration is very pronounced on the stability and the effectiveness of the different preparations based on the tested EOs.

The type of emulsifier seemed to have a significant effect (*p* < 0.05) on d_H_ and PDI (Figure 1). Indeed, the use of 1% wt. of a natural small-molecule surfactant, such as QS in formulation F9, did not lead to finer droplets (d_H_ = 339.2 ± 4.5 nm and PDI = 0.30 ± 0.03). However, in other studies, this surfactant was found to be effective for the formation of stable nanoemulsions with fine droplet size [50]. Similarly, Ozturk et al. [40] formed fine nanoemulsions (d_H_ < 150 nm) using 0.5% wt. QS and 2% wt. LEC for vitamin E encapsulation. The results presented in this work cannot, hence, be attributed to the surfactant but the oil phase. QS likely has a limited capability to reduce the extent of the Ostwald ripening of the EO.

#### 3.2.2. Effect of the Type of Stabilizer on the Nanoparticle Size Distribution

Only a few studies have addressed the encapsulation of antibacterial substances, such as EOs by low-energy methods using natural stabilizers [26,55,56,57,58]. In this work, the production of DSs (in the form of biopolymeric nanoparticles) was investigated by anti-solvent precipitation, using zein (ZN) in combination with different stabilizers. Polysaccharides are reported to increase the stability of DSs by increasing the viscosity of the aqueous phase under stress conditions [59,60]. The combination of proteins and polysaccharides is of interest due to their ability to form complex polyelectrolytes and the possibility of a Maillard reaction, stabilizing the layer around the EOs, through their physical entrapment [55,61]. Previously, ZN has been used to encapsulate thymol and carvacrol [62] and EOs from oregano and thyme [63]. ZN has proven to be a good material for the fabrication of DSs, such as nanoparticles [18,64,65,66,67].

Furthermore, ZN nanoparticles with sizes below 100 nm can be easily produced by antisolvent precipitation [27]; however, when ZN nanoparticles bind to polysaccharides, the droplet size increases [68,69,70,71]. The manufacture of ZN/polysaccharide nanoparticles generally involves two phases. First, the ZN molecules aggregate into nanoparticles accompanied by the encapsulation of hydrophobic compounds; then, the ZN nanoparticles bind to the polysaccharide molecules to form core–shell nanoparticles. In addition, some polysaccharides soluble in aqueous ethanol solution can aggregate with ZN to form complex nanoparticles and encapsulate bioactive compounds [56].

Anti-solvent precipitation has been applied to produce thyme EO nanoparticles, using ZN and ZN/polysaccharide complex to generate strong electrostatic repulsions between ZN nanoparticles as they form. Naked ZN nanoparticles exhibit strong mutual repulsion when the pH value is well below its isoelectric point (about 6.5). However, when pH is close to the isoelectric point, particle aggregation occurs due to hydrophobic attractions.

Figure 2 reports the d_H_ and PDI values for TC1 EO formulations F10–F13, with F10 corresponding to ZN-based nanoparticles, and F11–F13 to a combination of ZN with PEC, GA, and MD, respectively.

After anti-solvent precipitation, the colloidal dispersion formed by ZN alone (F10), presented particles with d_H_ = 75.8 ± 0.6 nm and PDI = 0.15 ± 0.03; this result was similar to that reported by Huang et al. [69]. However, the addition of PEC (F11) caused a significant increase in size, with d_H_ = 1444.0 ± 56.5 nm and PDI = 0.30 ± 0.02. These results differed from those previously reported about the encapsulation of curcumin in ZN/PEC nanoparticles by anti-solvent precipitation, which resulted in a d_H_ = 250 nm and PDI = 0.24 [68]. Furthermore, Huang et al. [69] developed ZN/PEC nanoparticles with d_H_ = 235 nm and PDI = 0.24 to encapsulate resveratrol, using a PEC concentration of 0.11% wt. The increase in particle size due to PEC can be related to two reasons: particle aggregation and shell formation [69].

Regarding the use of GA as a stabilizer (F12), d_H_ = 127.3 ± 1.2 nm and PDI of 0.15 ± 0.02 were observed. Previously, Chen and Zhong [72] prepared ZN nanoparticles stabilized with GA for the encapsulation of peppermint EO, with d_H_ = 160.7 ± 37.4 nm.

Remarkably, the ZN/MD formulation (F13) seemed to be the most effective for obtaining nanometric particles, resulting in d_H_ = 74.7 ± 1.7 nm and PDI = 0.14 ± 0.06. Kibici and Kahveci [73] tested the feasibility of using MD as a stabilizer to prepare fine and stable ZN-based nanoparticles.

In this work, extremely fine droplet sizes (d_H_ < 150 nm) were achieved using ZN/MD, ZN, and ZN/GA combinations, the emulsions with the smallest particle sizes being LEC and WP (d_H_ < 200 nm).

In general, our results show that the droplet size distribution of biopolymeric nanoparticles depends significantly (*p* < 0.05) on the stabilizer, with instability observed when PEC is used, whereas ZN/MD and ZN/GA combinations result in fine particles, comparable with ZN ones.

Similarly, the lowest PDI was obtained for DSs based on ZN, ZN/MD, and ZN/GA, indicating narrow size distributions, while the use of PEC caused greater size distribution. A wide particle size distribution could promote destabilization phenomena by coalescence mechanism, especially due to Ostwald ripening [74,75].

#### 3.2.3. Effect of Essential Oil Composition on the Size Distribution of the Delivery Systems

The EO composition showed a significant effect (*p* < 0.05) on the size distribution of DSs prepared with TC1 EO (Figure 1 and Figure 2), TC2 EO (Figure 3), and TA EO (Figure 4). Indeed, by comparing the DSs prepared with the three different EOs, TC2 and TA EOs presented the smallest particle sizes, with d_H_ = 59.9, 72.3, 113.4, 157.7, and 158.8 nm measured for the TC2 EO formulations based on ZN, ZN/MD, ZN/GA, WP, and LEC, respectively, and d_H_ = 65.5, 68.8, 113.2, 159.8, and 183.0 nm, respectively, for TA EO. TC1 EO, as discussed in the previous sections, exhibited the largest particle sizes (d_H_ = 75.8, 74.7, 127.3, 210.8, and 152.8 nm, respectively), although still in the nanometric range. These results, therefore, showed that the composition of EO plays an important role in affecting the size distribution of the DSs. In the specific case under investigation, the effect of size distribution could be attributed to the different carvacrol concentrations of the EOs. These results are in agreement with those of Mauriello et al. [28], who indicated that carvacrol-based nanoemulsions are severely affected by the occurrence of Ostwald ripening, with consequences on the observed size distributions.

### 3.3. ζ-Potential

The ζ-potential values, representing differences in the electric charges between the external ions and the fluid mass in suspension surrounding the nanoparticles, indicates the intensity of the repulsion forces between the nanoparticles. Large repulsive forces could prevent particle flocculation and aggregation, promoting stabilization of the colloidal system [76]. Additionally, in the case of ZN-based systems, the ζ-potential can help detect whether there are sufficient stabilizing polysaccharide molecules to cover the surface of the ZN nanoparticles, hence contributing to the determination of the optimal mass ratio of zein to polysaccharide [77]. In general, surfactant ions may be specifically adsorbed on the surface of a particle, leading, in the case of cationic surfactants, to a positively charged surface and, in the case of anionic surfactants, to a negatively charged surface (Malvern Instruments Limited, Malvern, UK, technical note, 2015).

In general, it can be observed that for non-ionic surfactants, such as Tween 80, a ζ-potential value close to neutral (0 mV) can be expected. In contrast, for lecithin-based or saponin-based emulsions, negative ζ-potential values are generally obtained [78,79]. In the case of zein-based systems, at their natural pH (the pH at which they are obtained, pH ≈ 4), zein-based particles exhibit a positive ζ-potential value (>30 mV). However, the addition of a stabilizer might significantly change the resulting pH value, especially if deposited on the particle surface through electrostatic interactions [80]. Therefore, the addition of GA and PEC caused an inversion of ζ-potential values to be negative. In contrast, in the case of MD, its interaction with zein is not based on electrostatic interaction but hydrophobic bonds, and therefore, particle ζ-potential is not altered, remaining positive.

Figure 5 reports the ζ-potential values of the DSs prepared with TC1 EO (formulations F1–F13). The ζ-potential ranged between −11.60 and 38.67 mV. It must be highlighted that the ζ-potential of nanoemulsions based on T80 was close to the neutrality and not of high quality, probably due to the nonionic nature of T80 and its surface interaction with TC1 EO. Similar observations are reported by Mauriello et al. [28].

The ζ-potential of LEC-based systems (F3 and F4) was −14.40 and −20.67 mV for 1.0% and 0.2% wt. LEC, with higher LEC concentrations increasing the ζ-potential value. Upon PEC addition, the ζ-potential gradually decreased (F5 and F6), reaching −12.13 and −13.17 mV for the concentrations of 0.66 and 0,16% wt. PEC, respectively. Without PEC, the observed ζ-potential values can be attributed to the surface coverage by LEC, characterized by negatively charged phosphate groups. Upon PEC addition to the system, the carboxyl groups of the pectin molecules lose protons and remain negatively charged at a pH of around 4.5, resulting in a decrease in ζ-potential [81].

Regarding the WP-based formulations (F7 and F8), the decrease in the concentration of WP and the addition of SO induced a decrease in ζ-potential, reaching the values of −11.60 and −13.33 mV, respectively.

In the case of the QS-based system (F9), the observed ζ potential was negative and relatively high (−17.30 mV), as reported in previous studies [50,82]. It is speculated that the negative surface charge density of QS-coated droplets is related to the presence of the carboxylic acid group in the chemical structure of QS. These highly negative charges provide strong electrostatic repulsion forces between the formed droplets, leading to a stable colloidal dispersion [83].

ZN-based nanoparticles (F10) exhibited at pH = 4 a ζ-potential of +35.53 mV. The addition of PEC resulted in the particles’ charge inversion, with a ζ-potential value of −34.00 mV. Zein is a highly hydrophobic protein with an isoelectric point at pI = 6.2 [84]. PEC is an anionic polysaccharide that has carboxyl groups with a dissociation constant (pKa) around pH 3.5 and, therefore, is characterized by a strong negative charge at pH 4 [85]. At such pH, ZN molecules exhibit a positive net charge, while PEC molecules exhibit a negative net charge, driving the adsorption of PEC molecules onto the surface of ZN particles by electrostatic attractions. The full coverage of the ZN particle surface by PEC molecules caused a negative net charge of the nanoparticles due to the pectin molecules forming an outer coating layer, with the anionic groups on PEC molecules exceeding the cationic groups on the ZN nanoparticles. For example, the encapsulation of curcumin by ZN/PEC through anti-solvent precipitation has shown particles with a ζ-potential of −28 mV [68].

The addition of GA to ZN particles (ZN/GA, formulation F12) induced a ζ-potential of −20.60 mV. similar to what was observed for PEC, the negatively charged GA adsorbs onto the positively charged ZN particles, leading to the inversion in the ζ-potential [86].

The addition of MD (ZN/MD, formulation F13) caused the ζ-potential value to increase to 38.67 mV, suggesting the stability of these DSs. An absolute value of ζ-potential greater than 30 mV was reported to indicate good physical stability due to the electrostatic repulsion of the particles [76].

By comparing these results with those obtained with other EOs, it appears that in the majority of the DSs investigated, TA EO-based formulations exhibited the highest ζ-potential values, followed by TC2 EO and then TC1 EO, although the dependence upon the EO type is not statistically significant (*p* < 0.05) (Table 3), with the resulting ζ-potential depending only on the DSs’ formulation.

### 3.4. Storage Stability

**Figure 8:**
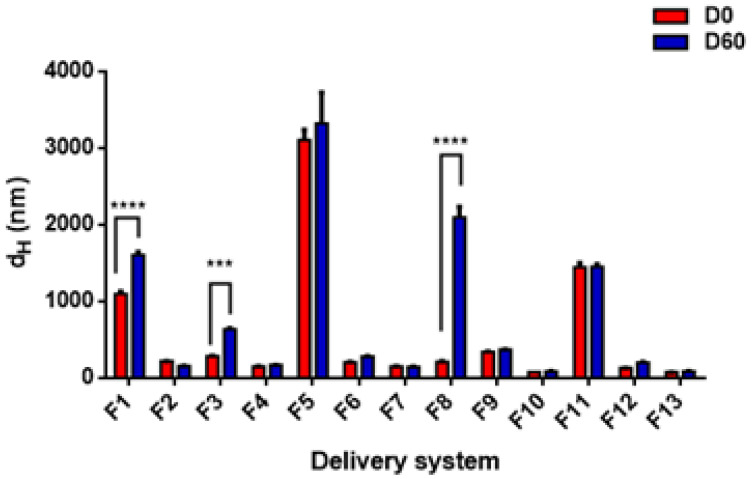
Effect of storage time on the stability of TC1 EO nanoemulsions (formulations F1–F9) and nanoparticles (formulations F10–F13). Statistical significance: *** *p* < 0.001 (very significant); **** *p* < 0.0001 (highly significant). Legend: day 0 (D0), day 60 (D60).

The long-term stability of colloidal systems is one of the most important factors in determining their shelf-life in commercial food and beverage applications [50,87]. This part of the study aims at comparing the stability of the different prepared DSs, using different emulsifiers and stabilizers, during storage at 4 °C for two months. Figure 8 shows the average droplet sizes (d_H_) at the beginning (day 0) and the end (day 60) of the storage period for all samples prepared with TC1 EO. Except for F1, F3, and F8 nanoemulsions, the droplet sizes did not change significantly during storage for the remaining DSs. A highly significant (*p* < 0.0001) increase in droplet size was detected for (F1) after 60 days of storage, increasing from 1099.0 ± 33.0 nm to 1609.0 ± 35.1 nm. The main reason for instability in this formulation, prepared with 2:1 EO/T80, was attributed to the large initial droplet size, which favored gravitational instability [88,89]. A less pronounced change (*p* < 0.001) was observed for F3, prepared with 1:1 EO/lecithin, where the size increased from 284.6 ± 3.7 nm on day 0 to 636.1 ± 13.6 nm on day 60. This increase in droplet size may be attributed to coalescence between droplets, not fully covered with lecithin. Furthermore, WP-based formulation F8 exhibited a strong tendency towards physical instability, with a highly significant increase from 210.8 ± 11.7 nm to 2095.0 ± 142.8 nm. Since WP proteins are characterized by a large molecular size, their adsorption on the surface of the droplets is slower than for small-molecule surfactants [43].

Droplet growth in the presence of high emulsifier concentration can be attributed to droplet coalescence and/or Ostwald ripening facilitated by emulsifier micelles [82]. However, these phenomena are not relevant in the case of biopolymeric nanoparticles, because of the physical entrapment of the EO by ZN to form a matrix-type structure, and the eventual addition of a polysaccharide to form an envelope layer on their surface. For example, formulations F10, F11, F12, and F13 did not undergo any significant change in particle size, as confirmed by the statistical analysis, and remained stable over the entire storage period. Moreover, the matrix-type structure might have also contributed to slowing down gravitational separation phenomena by reducing the density difference between the internal and external phases, in agreement with the results reported for peppermint oil encapsulated in ZN nanoparticles stabilized by GA adsorption [72].

**Figure 9:**
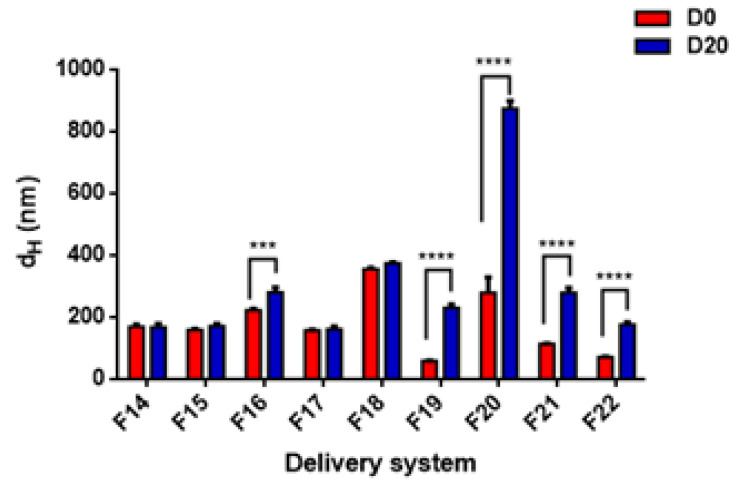
Effect of storage time on the stability of TC2 EO nanoemulsions (formulations F14–F18) and nanoparticles (formulations F19–F22). Statistical significance: *** *p* < 0.001 (very significant); **** *p* < 0.0001 (highly significant). Legend: day 0 (D0), day 60 (D60).

**Figure 10:**
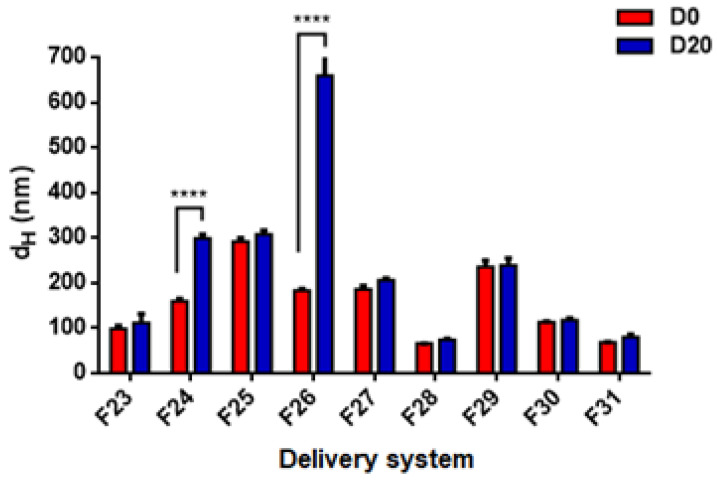
Effect of storage time on the stability of TA EO nanoemulsions (formulations F23–F27) and nanoparticles (formulations F28–F31). Statistical significance: **** *p* < 0.0001 (highly significant). Legend: day 0 (D0), day 60 (D60).

Regarding the stability of DSs prepared by TC2 and TA EOs, Figure 9 and Figure 10 show the best stability of several formulations after 20 days of storage, including both nanoemulsions (F14, F15, F17, F18, F23, F25, F27) and nanoparticles (F28, F29, F30, F31). In general, the DSs characterized by a large size at day 0 exhibit a strong tendency towards physical instability (mainly due to gravitational separation).

In addition, differences in EO composition, with different proportions of more or less soluble compounds, might affect the extent of the Ostwald ripening instability phenomena. For these reasons, nanoemulsions based on TC1 EO were generally scarcely stable, and different DSs prepared with TC2 EO (both nanoemulsions and nanoparticles) resulted in significant changes in size. More specifically, for TC2 EO, the LEC/PEC nanoemulsions and all the ZN-based nanoparticles showed a strong increase in size over time. Conversely, TA EO-based DSs presented only two unstable formulations, corresponding to LEC and WP-based nanoemulsions (F24 and F26, respectively), due to their different compositions, which are likely to be less prone to Ostwald ripening, and higher absolute values of ζ-potential, contributing to particle electrostatic repulsions. To conclude, the difference in the stability results of the three EOs is due to the difference in the carvacrol concentration of each EO. However, more detailed studies are necessary to fully elucidate this aspect.

### 3.5. Encapsulation Efficiency

Encapsulation efficiency (EE) is the percentage of EO substance effectively encapsulated in the DSs concerning the total EO loading. Due to a higher carvacrol content, TC1 EO was selected to be used in the assessment of the EE. The EE of TC1 EO was determined for the formulations F4, F6, and F8 (nanoemulsions) and F10, F11, F12, and F13 (nanoparticles). As shown in Table 4, the EE of ZN-based nanoparticles F10 (99.21%) was higher than that observed for the nanoemulsions formulations based on LEC, LEC/PEC, and WP. Furthermore, the stabilization of ZN particles through polysaccharides improved the trapping capacity of ZN. The ZN/GA-based formulation exhibited the highest EE (99.89%) followed by the ZN/MD (99.66%) and then the ZN/PEC (99.47%). Oliveira et al. (2018) have developed a biodegradable nano-pesticide using ZN as the carrier system for the botanical repellents Geraniol and Citronella, thus recording a higher EE (>90%). Moreover, a high EE of ZN-based nanoparticles was also found by Zhang et al. [90], which achieved values > 80% for thymol encapsulated in ZN nanoparticles, stabilized with sodium caseinate and chitosan hydrochloride. Similarly, Li et al. [77] reported an EE of 62.7% for polysaccharide-based nanoparticles.

### 3.6. Antioxidant Activity

The antioxidant activity of *Thymus capitatus* EO in bulk and encapsulated form (in different formulations) was tested through the FRAP test reduction of iron ions, with the results shown in Table 5. Generally, the FRAP method is especially suitable for antioxidant molecules of hydrophilic nature; however, it was also applied for TC1 EO encapsulated in water-based DSs. The results showed that bulk EO can supply enough Fe(II) by Fe(III) reduction to bind to TPTZ, with a resulting antioxidant activity corresponding to 57.7 µg AAE/mg of EO. The nanoencapsulation of thyme EO visibly improved its reducing power except for the nanoparticles containing GA (F12). These results differed from those previously reported by Ben Jemaa et al. [91], who demonstrated that the encapsulation of *T. capitatus* EO in SDS-based nanoemulsions decreases its antioxidant activity. The strong antioxidant activity of the formulations F6 and F11 could be attributed, in part, to pectin. Previous studies suggested that pectin can directly interact with oxidants and free radicals [92]. Similarly, the antioxidant capacity of the formulations F4 and F8 could be related to the presence of lecithin and whey protein since LEC and WP are endowed with antioxidant activity [93,94]. It should be noted that the retention of EO within the ZN nanostructure allows their dispersion in water with an improvement in their antioxidant effect, as reported by Wu et al. [62]. In a related study by Zhang et al. [95] ZN alone was able to inhibit free radicals.

By comparing the antioxidant activity of the formulations F12 and F13 the formulation based on MD exhibited a higher activity than GA. An earlier study by Sarabandi et al. [96] approved this behavior and found that the micro-encapsulation of eggplant peel extract with MD shows higher antioxidant activity than GA. Other authors have indicated that the protein fraction of GA promotes the Maillard reaction, leading to the formation of intermediate compounds capable of increasing the antioxidant activity of the final product [97]. However, the present study did not notice this behavior. It is likely that the GA strongly interacts with EO antioxidant components, reducing their resulting activities in the FRAP test. Further studies need to clarify this aspect. As shown in Section 3.7 (Antibacterial activity), the reduction in measured antioxidant activity for ZN/GA nanoparticles did not correspond to a reduction in antibacterial activity that increased.

Notably, the nanoemulsion-based systems (F4, F6, and F8) exhibited the highest antioxidant activity, in correspondence with the lowest EE values (Table 4). This can be explained by the fact that if EO components are not efficiently encapsulated, they are more readily available to interact with FRAP reactants. Moreover, the higher value than pure EO may be due to the antioxidant activity of the other nanoemulsion ingredients (e.g., LEC, PEC, SO). In contrast, for the nanoparticle-based systems, the EOs are physically entrapped in the DSs, with high EE and lower resulting antioxidant activity.

### 3.7. Antibacterial Activity

The well diffusion method was used to assess the antibacterial activity of the best TC1 EO formulations. When the antibacterial agent is encapsulated, the size of the inhibition zone (diameter) can provide information on the efficiency of the DS in releasing the EO. The size of the inhibition diameters of the nano-encapsulated EO varied between 12 and 35 mm, while the bulk EO varied between 9 and 28 mm (Table 6). Therefore, it could be deduced that the antimicrobial activity of TC1 EO encapsulated in ZN/GA (F11) or ZN/MD (F12) is always superior to that of bulk EO, suggesting an improvement in the transport mechanisms through the cell membrane of the target microorganisms. More specifically, ZN-based nanoparticles containing GA exhibited better antimicrobial activity than those containing MD. It can be speculated that the presence of GA might have increased the permeability of the cell membranes of bacteria, thus facilitating the entry of EO into the cells, allowing the release of EO in the aqueous phase, and thus improving its antimicrobial activity. The difference in antimicrobial activity of the different DSs may be related to differences in the inherent antimicrobial activity of the stabilizers, or in their abilities to modify cell membrane permeability. MD does not have any antimicrobial activity, whereas GA is reported to exhibit a certain biological activity, which enhances the antimicrobial activity of the nanoencapsulated EOs [98]. This was supported by numerous studies, including that of Ali et al. [99], and can be attributed to the presence of –OH groups with remarkable effects on protein-cell binding, enzyme inactivation, and DNA replication of microorganisms [100]. It has been proved that antimicrobial encapsulation can lead to the development of microcapsules capable of inhibiting the growth of pathogenic microorganisms [98]. It was also reported that the antibacterial effects of encapsulated substances differ depending on the coating material used for encapsulation [101].

The results of this study confirmed that the antibacterial activity of DSs is more pronounced against the bacterial strains of *E. coli, S. typhimurium*, and *L. monocytogenes*. Therefore, the effect of nanoencapsulated EO on the antibacterial activity depends on the target microorganism. These results are in agreement with those of Moghimi et al. [13], who showed that sage oil nanoemulsions exhibit potent antibacterial activity against *S. typhimurium* and *E. coli*, as compared to bulk oil. Additionally, Moghimi et al. [102] showed that nanoemulsions of *Thymus daenensis* EO present better antimicrobial activity than bulk EO against *E. coli*. In this same context, the studies by Wu et al. [62] reported that the retention of EOs within the ZN nanostructure allows their dispersion in water with an improvement in their antimicrobial effect. Several other studies have also demonstrated an improvement in the antibacterial activity of EOs when they are nanoencapsulated [90,103,104,105]. However, some studies showed that there is no change in the antimicrobial activity of EOs when emulsified [23,51,106]. A possible explanation for these differences could be related to the incorporation of carrier oil such as corn oil and soybean oil, which may reduce the antibacterial activity of emulsions by acting as a high-affinity solvent for EOs, reducing the amount of EO in equilibrium with the aqueous phase and hence making it available to interact with bacteria [23,28,51,107].

The present study is the first report describing the formulation of nanometric DSs containing *T. capitatus* EO, using low emulsifier and/or natural stabilizer concentrations, without incorporation of carrier oil, and showing long-term stability with significant biological effects. It should be mentioned that comparison with the data from the literature is not simple, because of the different microbial strains considered, the different types of emulsifier/stabilizer along with the different experimental procedures used, in particular, to evaluate the microbial viability.

Based on aromatogram tests, in conjunction with stability and antioxidant characterization, it can be inferred that in the case of *T. capitatus* EO, the optimal DS formulation is based on biopolymeric nanoparticle colloidal suspensions containing 0.4% wt. EO and formulated with 1% wt. ZN and 0.33% wt. MD or GA. These results have important implications for the formulation of natural antimicrobial DSs suitable for use in food, pharmaceutical, and cosmetic products. Additionally, future studies are recommended to address the issues related to masking undesirable taste and aroma (pronounced/intense) features of EOs, for application in a wide range of food products.

## 4. Conclusions

This work introduces the diverse types of nanoencapsulation systems that were investigated for the delivery of thyme EOs (*Thymus capitatus* and *Thymus algeriensis*) as antimicrobial agents to be used in food systems. The different EOs tested exhibited significantly different compositions, depending on when and where they are harvested. In particular, *T. capitatus* EO, harvested in July 2018 in Tunisia, were extremely rich in carvacrol. The different EOs were encapsulated in nanoemulsion and nanoparticle colloidal delivery systems (DSs) and formulated using different emulsifiers and natural stabilizers. The results showed that carvacrol, the major component of EOs, plays an important role in the formation of stable DSs, especially in terms of the possibility to obtain a nanometric size system, with sufficient stability over time. The most promising formulation for TC1 EO was the colloidal nanoparticle suspension based on zein and maltodextrins (ZN/MD), characterized by fine particle size, reduced polydispersity (PDI < 0.2), and the absolute value of ζ-potential > 30 mV; it also showed an encapsulation efficiency >99%, a remarkable antioxidant, and antibacterial activity. This optimal formulation has been achieved by using reduced amounts of natural stabilizers and it can be beneficial for food and beverage manufacturers to use natural ingredients in their products. The antibacterial activity tests against pathogenic bacteria showed that the most stable DS formulations contribute to enhancing the EOs’ antibacterial activity, demonstrating that thyme EOs can be used as a preservative agent in meat products to extend the food shelf-life. However, several challenges are addressed to exploiting the EO DSs as part of a food preservation strategy. For example, future studies should demonstrate the persistence of the activity of EOs DSs over time and under different food manufacture and storage conditions, as well as the capability of masking, at least partly, the flavor and taste alterations related to the use of these systems in foods. Moreover, it is suggested that a morphological characterization of the delivery systems is carried out to contribute to identifying their application as food preservatives. In general, zein-based nanoparticles exhibit excellent compatibility with protein-based foods, such as pasta, baked products, meat, and dairy products, as well as in protein-based edible coatings. In contrast, emulsion-based systems find perspective application in beverages, as well as in fat-rich foods (e.g., gravies, creams, dressings, mayonnaise, ice cream, etc.). Therefore, the final application depends not only on the intrinsic (as measured in vitro) properties of the delivery system but also on its compatibility with the food system.

## Figures and Tables

**Table 1:** Formulations of delivery systems of essential oils of TC1 (*Thymus capitatus,* harvested in July), TC2 (*Thymus capitatus,* harvested in April), and TA (*Thymus algeriensis,* harvested in April).Abbreviations: HPH (High-Pressure Homogenization), T80 (Tween 80), LEC (Lecithin), PEC (Pectin), WP (Whey proteins), SO (Sunflower oil), QS (Quillaja saponin), ZN (Zein), GA (Gum arabic), MD (Maltodextrin).

Formulation	EO %	Emulsifiers/Stabilizers	EO:Emulsifier Ratio	Methods	Conditions
F1	TC1 (1% wt.)	0.5% wt. T80	2:1	HPH	100 MPa, 5 passes
F2	TC1 (1.5% wt.)	1.5% wt. T80	1:1	HPH	100 MPa, 5 passes
F3	TC1 (1.0% wt.)	1.0% wt. LEC	1:1	HPH	100 MPa, 5 passes
F4	TC1 (0.1% wt.)	0.2% wt. LEC	1:2	HPH	100 MPa, 5 passes
F5	TC1 (1.0% wt.)	1.0% wt. LEC + 0.66% wt. PEC	1:1	HPH	100 MPa, 5 passes
F6	TC1 (0.1% wt.)	0.2% wt. LEC + 0.166% wt. PEC	1:2	HPH	100 MPa, 5 passes
F7	TC1 (1% wt.)	2.0% wt. WP	1:2	HPH	100 MPa, 5 passes
F8	TC1 (0.1% wt.)	0.1% wt. SO + 0.4% wt. WP	1:4	HPH	100 MPa, 5 passes
F9	TC1 (0.1% wt.)	0.1% wt. QS	1:1	HPH	100 MPa, 5 passes
F10	TC1 (0.4% wt.)	1.0% wt. ZN	1:2.5	Solvent diffusion	Antisolvent precipitation of ethanol solution in water, ethanol removal under reduced pressure
F11	TC1 (0.4% wt.)	1.0% wt. ZN + 0.33% wt. PEC	1:2.5	Solvent diffusion	Antisolvent precipitation of ethanol solution in aqueous solution, ethanol removal under reduced pressure
F12	TC1 (0.4% wt.)	1.0% wt. ZN+ 0.33% wt. GA	1:2.5	Solvent diffusion	Antisolvent precipitation of ethanol solution in aqueous solution, ethanol removal under reduced pressure
F13	TC1 (0.4% wt.)	1.0% wt. ZN + 0.33% wt. MD	1:2.5	Solvent diffusion	Antisolvent precipitation of ethanol solution in aqueous solution, ethanol removal under reduced pressure
F14	TC2 (1.5% wt.)	1.5% wt. T80	1:1	HPH	100 MPa, 5 passes
F15	TC2 (0.1% wt.)	0.2% wt. LEC	1:2	HPH	100 MPa, 5 passes
F16	TC2 (0.1% wt.)	0.2% wt. LEC + 0.166% wt. PEC	1:2	HPH	100 MPa, 5 passes
F17	TC2 (0.1% wt.)	0.1% wt. SO. 0.4% wt. WP	1:1	HPH	100 MPa, 5 passes
F18	TC2 (0.1% wt.)	0.1% wt. QS	1:1	HPH	100 MPa, 5 passes
F19	TC2 (0.4% wt.)	1.0% wt. ZN	1:2.5	Solvent diffusion	Antisolvent precipitation of ethanol solution in water, ethanol removal under reduced pressure
F20	TC2 (0.4% wt.)	1.0% wt. ZN + 0.33% wt. PEC	1:2.5	Solvent diffusion	Antisolvent precipitation of ethanol solution in aqueous solution, ethanol removal under reduced pressure
F21	TC2 (0.4% wt.)	1.0% wt. ZN + 0.33% wt. GA	1:2.5	Solvent diffusion	Antisolvent precipitation of ethanol solution in aqueous solution, ethanol removal under reduced pressure
F22	TC2 (0.4% wt.)	1.0% wt. ZN + 0.33% wt. MD	1:2.5	Solvent diffusion	Antisolvent precipitation of ethanol solution in aqueous solution, ethanol removal under reduced pressure
F23	TA (1.5% wt.)	1.5% wt. T80	1:1	HPH	100 MPa, 5 passes
F24	TA (0.1% wt.)	0.2% wt. LEC	1:2	HPH	100 MPa, 5 passes
F25	TA (0.1% wt.)	0.2% wt. LEC + 0.17% wt. PEC	1:2	HPH	100 MPa, 5 passes
F26	TA (0.1% wt.)	0.1% wt. SO. 0.4% wt. WP	1:1	HPH	100 MPa, 5 passes
F27	TA (0.1% wt.)	0.1% wt. QS	1:1	HPH	100 MPa, 5 passes
F28	TA (0.4% wt.)	1.0% wt. ZN	1:2.5	Solvent diffusion	Antisolvent precipitation of ethanol solution in water, ethanol removal under reduced pressure
F29	TA (0.4% wt.)	1.0% wt. ZN + 0.33% wt. PEC	1:2.5	Solvent diffusion	Antisolvent precipitation of ethanol solution in aqueous solution, ethanol removal under reduced pressure
F30	TA (0.4% wt.)	1.0% wt. ZN + 0.33% wt. GA	1:2.5	Solvent diffusion	Antisolvent precipitation of ethanol solution in aqueous solution, ethanol removal under reduced pressure
F31	TA (0.4% wt.)	1.0% wt. ZN + 0.33% wt. MD	1:2.5	Solvent diffusion	Antisolvent precipitation of ethanol solution in aqueous solution, ethanol removal under reduced pressure

**Table 2:** Chemical composition of essential oils is reported as the area of the peak of the different compounds, as determined through GC-MS analysis. In the case of carvacrol, also the concentration is reported, through comparison with a carvacrol standard.

Constituents	RT	TC1	TC2	TA
α-thujene	6.63	33,507,780	3,669,328	8,398,099
α -pinene	6.87	17,689,954	1,576,497	572,237,616
Camphene	7.46	-	-	170,933,287
Sabinene	8.30	-	-	17,786,949
β-pinene	8.48	-	-	89,061,284
β -myrcene	8.97	47,816,381	2,519,797	12,826,455
terpinolene	10.05	63,561,201	4,254,635	11,792,738
β –cimene	10.36	350,814,610	26,462,214	55,120,443
Limonene	10.58	-	-	62,588,570
Eucalyptol	10.85	-	-	493,032,621
β –ocimene	11.38	-	-	47,470,846
γ-terpinene	11.83	264,721,160	19,234,266	33,772,823
Linalool	13.84	46,446,149	2,001,442	53,653,939
Endo-borneol	17.08	19,797,940	2,082,693	124,024,724
Terpinen-4-ol	17.53	34,429,457	1,727,441	34,698,306
α –terpineol	18.31	-	-	62,134,380
D-carvone	20.43	-	-	6,042,611
Borneol acetate	22.02	-	-	85,221,967
Thymol	22.26	19,714,946	643,531	-
Carvacrol	22.55	2,925,213,353 (907 g/L)	161,230,188 (50.9 g/L)	205,213,346 (63.6 g/L)
Caryophyllene	26.46	98,406,001	2,493,947	63,494,548
Caryophyllene oxide	29.27	27,494,870	654,006	201,335,934

RT: retention index, TC1: *Thymus capitatus* harvested in July 2018, TC2: *Thymus capitatus* harvested in April 2018, TA: *Thymus algeriensis* harvested in April 2018.

**Table 3:** Hydrodynamic diameter d_H_, polydispersity index PDI, and ζ-potential of the tested delivery systems for essential oils.

Formulations	d_H_ [nm]	PDI [−]	ζ-Potential [mV]
F1	1099.0 ± 33.0 ^C,a^	0.41 ± 0.04 ^B,a^	−3.28 ± 3.06 ^E,a^
F2	218.4 ± 2.4 ^GH,a^	0.37 ± 0.03 ^BC,a^	−2.23 ± 0.89 ^E,a^
F3	284.6 ± 3.8 ^EF,a^	0.32 ± 0.04 ^CD,a^	−14.40 ± 0.61 ^FG,a^
F4	152.8 ± 2.2 ^JKLM,a^	0,21 ± 0.01 ^GHIJK,a^	−20.67 ± 0.90 ^IJ,a^
F5	3108.0 ± 132.6 ^A,a^	0.22 ± 0.06 ^FGHIJ,a^	−12.13 ± 0.91 ^F,a^
F6	206.6 ± 1.7 ^GHIJ,a^	0.22 ± 0.01 ^FGHIJ,a^	−13.17 ± 0.81 ^F,a^
F7	210.8 ± 11.7 ^GHI,a^	0.21 ± 0.01 ^DEF,a^	−11.60 ± 0.61 ^F,a^
F8	152.2 ± 1.2 ^KLM,a^	0.28 ± 0.05 ^GHIJKL,a^	−13.33 ± 0.96 ^F,a^
F9	339.2 ± 4.5 ^D,a^	0.30 ± 0.03 ^DE,a^	−17.30 ± 0.89 ^H,a^
F10	75.8 ± 0.6 ^ON,a^	0.15 ± 0.03 ^JKLM,a^	35.53 ± 1.92 ^D,a^
F11	1444.0 ± 56.5 ^B,a^	0.30 ± 0.02 ^DE,a^	−16.10 ± 1.21 ^GH,a^
F12	127.3 ± 1.2 ^LMN,a^	0.15 ± 0.02 ^JKLM,a^	−20.60 ± 2.01 ^IJ,a^
F13	74.7 ± 1.7 ^O,a^	0.14 ± 0.06 ^KLMM,a^	38.67 ± 0.72 ^C,a^
F14	169.0 ± 8.4 ^HIJKL,b^	0.23 ± 0.01 ^EFGHI,a^	−2.82 ± 0.40 ^E,a^
F15	158.8 ± 3.8 ^IJKLM,b^	0.32 ± 0.03 ^CD,a^	−20.10 ± 1.95 ^IJ,a^
F16	222.6 ± 5.4 ^G,b^	0.16 ± 0.00 ^IJKLM,a^	−16.30 ± 0.95 ^GH,a^
F17	157.7 ± 2.6 ^IJKLM,b^	0.19 ± 0.01 ^GHIJKL,a^	−12.67 ± 1.07 ^F,a^
F18	355.2 ± 4.8 ^D,b^	0.25 ± 0.02 ^DEFG,a^	−17.87 ± 1.78 ^IH,a^
F19	59.9 ± 0.8 ^O,b^	0.25 ± 0.02 ^JKLM,a^	37.63 ± 3.07 ^CD,a^
F20	279.3 ± 50.1 ^EF,b^	0.49 ± 0.11 ^A,a^	−27.80 ± 1.65 ^K,a^
F21	113.4 ± 1.9 ^MNO,b^	0.11 ± 0.02 ^M,a^	−22.90 ± 1.54 ^J,a^
F22	72.3 ± 0.5 ^O,b^	0.13 ± 0.01 ^LM,a^	49.30 ± 0.30 ^A,a^
F23	98.4 ± 7.1 ^NO,b^	0.42 ± 0.09 ^B,a^	−3.99 ± 0.11 ^E,a^
F24	159.8 ± 4.8 ^IJKLM,b^	0.21 ± 0.01 ^FGHIJK,a^	−18.23 ± 0.21 ^HI,a^
F25	291.7 ± 7.6 ^E,b^	0.25 ± 0.05 ^DEFGH,a^	−17.03 ± 1.50 ^GH,a^
F26	183.0 ± 3.6 ^GHIJK,b^	0.31 ± 0.04 ^CD,a^	−12.40 ± 0.53 ^F,a^
F27	185.6 ± 8.5 ^GHIJK,b^	0.18 ± 0.00 ^GHIJKL,a^	−21.30 ± 2.0 ^J,a^
F28	65.5 ± 0.7 ^O,b^	0.17 ± 0.01 ^IJKLM,a^	46.97 ± 1.72 ^A,a^
F29	236.2 ± 13.2 ^FG,b^	0.30 ± 0.05 ^DE,a^	−29.50 ± 1.65 ^K,a^
F30	113.2 ± 1.2 ^MNO,b^	0.16 ± 0.02 ^JKLM,a^	−32.07 ± 1.70 ^B,a^
F31	68.8 ± 0.5 ^O,b^	0.18 ± 0.02 ^HIJKLM,a^	42.20 ± 3.47 ^L,a^

Values are mean ± SE of three replicates. Different letters within the same column indicate significant differences (one-way ANOVA and Duncan test, *p* < 0.05); capital letters: comparison among formulations, lowercase letter: comparison among EOs (TC1, TC2, and TA).

**Table 4:** Encapsulation efficiency (EE) of selected formulations.

Formulations	EE (%)
F4	6.64
F6	0.88
F8	4.63
F10	99.21
F11	99.47
F12	99.89
F13	99.66

**Table 5:** Antioxidant activity of essential oil of *Thymus capitatus* (TC1) encapsulated in different formulations.

Samples	Antioxidant Activity (µg AAE/mg)
F4	80.21 ± 12.83
F6	108.28 ± 4.78
F8	115.79 ± 1.93
F10	67.17 ± 0.58
F11	84.04 ± 3.93
F12	12.26 ± 0.93
F13	60.69 ± 1.25
TC1 EO	57.69 ± 1.54

Results expressed in EAA (equivalent of ascorbic acid)/mg of loaded EO.

**Table 6:** Aromatograms for different bacteria exposed to the most promising *T. capitatus* (TC1) EO nanoparticles (F12 and F13) in comparison with pure EO. The diameters of the inhibition zones are given in mm.

	*T. capitatus EO* (TC1)	ZN/GA (F12)	ZN/MD (F13)	Significance
*Pseudomonas aeruginosa* ATCC 27853	12 ± 1	16 ± 2	14 ± 2	**
*Staphylococcus aureus* ATCC 25923	9 ± 2	14 ± 1	12 ± 1	**
*Escherichia coli ATCC* 25922	28 ± 1	35 ± 1	33 ± 2	***
*Salmonella typhimurium ATCC* 1402	19 ± 1	32 ± 2	29 ± 2	***
*Listeria monocytogenes ATCC 19118*	17 ± 1	28 ±1	25 ±1	***

Statistical significance: ** *p* < 0.01 (significant); ***p < 0.001 (very significant).

## Data Availability

The data presented in this study are available on request from the corresponding author.

## References

[1] Ho C.-L., Eugene I., Wang C., Su Y.-C. (2009). Essential oil compositions and bioactivities of the various parts of *Cinnamomum camphora* Sieb. var. linaloolifera Fujuta. Q. J. For. Res..

[2] Modarresi Chahardehi A., Ibrahim D., Fariza Sulaiman S. (2010). Antioxidant, Antimicrobial Activity and Toxicity Test of Pilea microphylla. Int. J. Microbiol..

[3] Spréa R.M., Fernandes Â., Calhelha R.C., Pereira C., Pires T.C.S.P., Alves M.J., Canan C., Barros L., Amaral J.S., Ferreira I.C.F.R. (2020). Chemical and bioactive characterization of the aromatic plant Levisticum officinale WDJ Koch: A comprehensive study. Food Funct..

[4] Bhavaniramya S., Vishnupriya S., Al-Aboody M.S., Vijayakumar R., Baskaran D. (2019). Role of essential oils in food safety: Antimicrobial and antioxidant applications. Grain Oil Sci. Technol..

[5] Pandey A.K., Kumar P., Singh P., Tripathi N.N., Bajpai V.K. (2017). Essential oils: Sources of antimicrobials and food preservatives. Front. Microbiol..

[6] Fathi M., Vinceković M., Jurić S., Viskić M., Režek Jambrak A., Donsì F. (2019). Food-Grade Colloidal Systems for the Delivery of Essential Oils. Food Rev. Int..

[7] Jayari A., El Abed N., Jouini A., Mohammed Saed Abdul-Wahab O., Maaroufi A., Ben Hadj Ahmed S. (2018). Antibacterial activity of Thymus capitatus and Thymus algeriensis essential oils against four food-borne pathogens inoculated in minced beef meat. J. Food Saf..

[8] Khorshidian N., Yousefi M., Khanniri E., Mortazavian A.M. (2018). Potential application of essential oils as antimicrobial preservatives in cheese. Innov. Food Sci. Emerg. Technol..

[9] Gray J.A., Chandry P.S., Kaur M., Kocharunchitt C., Bowman J.P., Fox E.M. (2018). Novel Biocontrol Methods for Listeria monocytogenes Biofilms in Food Production Facilities. Front. Microbiol..

[10] Hyldgaard M., Mygind T., Meyer R.L. (2012). Essential oils in food preservation: Mode of action, synergies, and interactions with food matrix components. Front. Microbiol..

[11] Jayari A., Jouini A., Boukhris H., Hamrouni S., Damergi C., Ben Hadj Ahmed S., Maaroufi A. (2021). Essential Oils from Thymus capitatus and Thymus algeriensis as Antimicrobial Agents to Control Pathogenic and Spoilage Bacteria in Ground Meat. J. Food Qual..

[12] Rudy M., Kucharyk S., Duma-Kocan P., Stanisławczyk R., Gil M. (2020). Unconventional Methods of Preserving Meat Products and Their Impact on Health and the Environment. Sustainability.

[13] Moghimi R., Aliahmadi A., McClements D.J., Rafati H. (2016). Investigations of the effectiveness of nanoemulsions from sage oil as antibacterial agents on some food borne pathogens. LWT Food Sci. Technol..

[14] Ali H., Al-Khalifa A.R., Aouf A., Boukhebti H., Farouk A. (2020). Effect of nanoencapsulation on volatile constituents, and antioxidant and anticancer activities of Algerian Origanum glandulosum Desf. essential oil. Sci. Rep..

[15] Jemaa M.B., Falleh H., Serairi R., Neves M.A., Snoussi M., Isoda H., Nakajima M., Ksouri R. (2018). Nanoencapsulated Thymus capitatus essential oil as natural preservative. Innov. Food Sci. Emerg..

[16] Donsi F., Ferrari G. (2016). Essential oil nanoemulsions as antimicrobial agents in food. J. Biotechnol..

[17] Maryam I., Huzaifa U., Hindatu H., Zubaida S. (2015). Nanoencapsulation of essential oils with enhanced antimicrobial activity: A new way of combating antimicrobial Resistance. Pharmacogn. Phytochem..

[18] Cheng C.J., Ferruzzi M., Jones O.G. (2019). Fate of lutein-containing zein nanoparticles following simulated gastric and intestinal digestion. Food Hydrocoll..

[19] McClements D., Rao J. (2011). Food-grade nanoemulsions: Formulation, fabrication, properties, performance, biological fate, and potential toxicity. Crit. Rev. Food Sci..

[20] Solans C., Izquierdo P., Nolla J., Azemar N., Garcia-Celma M.J. (2005). Nano-emulsions. Curr. Opin. Colloid Interface Sci..

[21] Quintão F.J.O., Tavares R.S.N., Vieira-Filho S.A., Souza G.H.B., Santos O.D.H. (2013). Hydroalcoholic extracts of Vellozia squamata: Study of its nanoemulsions for pharmaceutical or cosmetic applications. Rev. Bras. Farmacogn..

[22] Rao J., McClements D.J. (2012). Lemon oil solubilization in mixed surfactant solutions: Rationalizing microemulsion & nanoemulsion formation. Food Hydrocoll..

[23] Chang Y., McLandsborough L., McClements D.J. (2012). Physical properties and antimicrobial efficacy of thyme oil nanoemulsions: Influence of ripening inhibitors. J. Agric. Food Chem..

[24] Donsì F., Jafari S.M., McClements D.J. (2018). Applications of Nanoemulsions in Foods. Nanoemulsions.

[25] Fathi M., Donsi F., McClements D.J. (2018). Protein-based delivery systems for the nanoencapsulation of food ingredients. Compr. Rev. Food Sci. Food Saf..

[26] Donsi F., Voudouris P., Veen S.J., Velikov K.P. (2017). Zein-based colloidal particles for encapsulation and delivery of epigallocatechin gallate. Food Hydrocoll..

[27] Gali L., Bedjou F., Ferrari G., Donsì F. (2022). Formulation and characterization of zein/gum arabic nanoparticles for the encapsulation of a rutin-rich extract from *Ruta chalepensis* L.. Food Chem..

[28] Mauriello E., Ferrari G., Donsi F. (2021). Effect of formulation on properties, stability, carvacrol release and antimicrobial activity of carvacrol emulsions. Colloids Surf. B Biointerfaces.

[29] Fernández K., Roeckel M., Canales E., Dumont J. (2017). Modeling of the nanoparticles absorption under a gastrointestinal simulated ambient condition. AAPS PharmSciTech.

[30] Triyono K., Suhartatik N., Wulandari Y.W. (2018). Nanoencapsulating of kaffir lime oil with coacervation method using Arabic gum and maltodextrin as encapsulant. Int. J. Food Nutr. Sci..

[31] Guo Y., Jauregi P. (2018). Protective effect of β-lactoglobulin against heat induced loss of antioxidant activity of resveratrol. Food Chem..

[32] Cintas L.M., Rodriguez J.M., Fernandez M.F., Sletten K., Nes I.F., Hernandez P.E., Holo H. (1995). Isolation and characterization of pediocin L50, a new bacteriocin from Pediococcus acidilactici with a broad inhibitory spectrum. Appl. Environ. Microbiol..

[33] Hassan M.E., Hassan R.R., Diab K.A., El-Nekeety A.A., Hassan N.S., Abdel-Wahhab M.A. (2021). Nanoencapsulation of thyme essential oil: A new avenue to enhance its protective role against oxidative stress and cytotoxicity of zinc oxide nanoparticles in rats. Environ. Sci. Pollut. Res..

[34] El Abed N., Kaabi B., Smaali M.I., Chabbouh M., Habibi K., Mejri M., Marzouki M.N., Ben Hadj Ahmed S. (2014). Chemical Composition, Antioxidant and Antimicrobial Activities of Thymus capitata Essential Oil with Its Preservative Effect against Listeria monocytogenes Inoculated in Minced Beef Meat. Evid. Based Complement. Altern. Med..

[35] Gonçalves J.C.R., de Meneses D.A., de Vasconcelos A.P., Piauilino C.A., Almeida F.R.D.C., Napoli E.M., de Araújo D.A.M. (2017). Essential oil composition and antinociceptive activity of Thymus capitatus. Pharm. Biol..

[36] Nikolic M., Glamočlija J., Ferreira I.C., Calhelha R.C., Fernandes Â., Marković T., Soković M. (2014). Chemical composition, antimicrobial, antioxidant and antitumor activity of *Thymus serpyllum* L., Thymus algeriensis Boiss. and Reut and *Thymus vulgaris* L. essential oils. Ind. Crops Prod..

[37] Dob T., Dahmane D., Benabdelkader T., Chelghoum C. (2006). Studies on the essential oil composition and antimicrobial activity of Thymus algeriensis Boiss. et Reut. Int. J. Aromather..

[38] Gupta A., Eral H.B., Hatton T.A., Doyle P.S. (2016). Nanoemulsions: Formation, properties and applications. Soft Matter.

[39] Zhang R., Zhang Z., McClements D.J. (2020). Nanoemulsions: An emerging platform for increasing the efficacy of nutraceuticals in foods. Colloids Surf. B Biointerfaces.

[40] Ozturk B., Argin S., Ozilgen M., McClements D.J. (2015). Formation and stabilization of nanoemulsion-based vitamin E delivery systems using natural biopolymers: Whey protein isolate and gum arabic. Food Chem..

[41] Donsi F., Ferrari G. (2019). Effect of Nanoemulsion Formulation on Permeation of Essential Oils Through Biological Membranes. Chem. Eng. Trans..

[42] Barradas T.N., Holanda e Silva K.G.D. (2020). Nanoemulsions as optimized vehicles for essential oils. Sustainable Agriculture Reviews 44.

[43] Adjonu R., Doran G., Torley P., Agboola S. (2014). Whey protein peptides as components of nanoemulsions: A review of emulsifying and biological functionalities. J. Food Eng..

[44] Kuhn K.R., Cunha R.L. (2012). Flaxseed oil-whey protein isolate emulsions: Effect of high pressure homogenization. J. Food Eng..

[45] Yerramilli M., Ghosh S. (2017). Long-term stability of sodium caseinate-stabilized nanoemulsions. J. Food Sci. Technol..

[46] Tastan Ö., Ferrari G., Baysal T., Donsi F. (2016). Understanding the effect of formulation on functionality of modified chitosan films containing carvacrol nanoemulsions. Food Hydrocoll..

[47] Charoen R., Jangchud A., Jangchud K., Harnsilawat T., Naivikul O., McClements D.J. (2011). Influence of biopolymer emulsifier type on formation and stability of rice bran oil in water emulsions: Whey protein, gum arabic, and modified starch. J. Food Sci..

[48] Qian C., McClements D.J. (2011). Formation of nanoemulsions stabilized by model food-grade emulsifiers using high-pressure homogenization: Factors affecting particle size. Food Hydrocoll..

[49] Jafari S.M., He Y., Bhandari B. (2007). Effectiveness of encapsulating biopolymers to produce sub-micron emulsions by high energy emulsification techniques. Food Res. Int..

[50] Yang Y., Leser M.E., Sher A.A., McClements D.J. (2013). Formation and stability of emulsions using a natural small molecule surfactant: Quillaja saponin (Q-Naturale^®^). Food Hydrocoll..

[51] Ziani K., Chang Y., McLandsborough L., McClements D.J. (2011). Influence of surfactant charge on antimicrobial efficacy of surfactant-stabilized thyme oil nanoemulsions. J. Agric. Food Chem..

[52] Klang V., Valenta C. (2011). Lecithin-based nanoemulsions. J. Drug Deliv. Sci. Technol..

[53] McClements D.J. (1974). Ultrasonic determination of depletion flocculation in oil-in-water emulsions containing a non-ionic surfactant. Colloids Surf. A Physicochem. Eng. Asp..

[54] Mazarei Z., Rafati H. (2019). Nanoemulsification of Satureja khuzestanica essential oil and pure carvacrol; comparison of physicochemical properties and antimicrobial activity against food pathogens. LWT.

[55] Farshi P., Tabibiazar M., Ghorbani M., Mohammadifar M., Amirkhiz M.B., Hamishehkar H. (2019). Whey protein isolate-guar gum stabilized cumin seed oil nanoemulsion. Food Biosci..

[56] Li M., Yu M. (2020). Development of a nanoparticle delivery system based on zein/polysaccharide complexes. J. Food Sci..

[57] Luis A.I.S., Campos E.V.R., de Oliveira J.L., Guilger-Casagrande M., de Lima R., Castanha R.F., de Castro V., Fraceto L.F. (2020). Zein Nanoparticles Impregnated with Eugenol and Garlic Essential Oils for Treating Fish Pathogens. ACS Omega.

[58] Oliveira J.L.D., Campos E.V.R., Pereira A.E.S., Pasquoto T., Lima R., Grillo R., Andrade D.J.D., Santos F.A.D., Fraceto L.F. (2018). Zein nanoparticles as eco-friendly carrier systems for botanical repellents aiming sustainable agriculture. J. Agric. Food Chem..

[59] Dickinson E. (2009). Hydrocolloids as emulsifiers and emulsion stabilizers. Food Hydrocoll..

[60] Zhang J., Bing L., Reineccius G.A. (2015). Formation, optical property and stability of orange oil nanoemulsions stabilized by *Quallija saponins*. LWT Food Sci. Technol..

[61] Sonu K.S., Mann B., Sharma R., Kumar R., Singh R. (2018). Physico-chemical and antimicrobial properties of d-limonene oil nanoemulsion stabilized by whey protein-maltodextrin conjugates. J. Food Sci. Technol..

[62] Wu Y., Luo Y., Wang Q. (2012). Antioxidant and antimicrobial properties of essential oils encapsulated in zein nanoparticles prepared by liquid–liquid dispersion method. LWT Food Sci. Technol..

[63] Parris N., Cooke P.H., Hicks K.B. (2005). Encapsulation of essential oils in zein nanospherical particles. J. Agric. Food Chem..

[64] Da Rosa C.G., De Melo A.P.Z., Sganzerla W.G., Machado M.H., Nunes M.R., Maciel M.V.D.O.B., Cleber B.F., Barreto P.L.M. (2020). Application in situ of zein nanocapsules loaded with Origanum vulgare Linneus and Thymus vulgaris as a preservative in bread. Food Hydrocoll..

[65] Gagliardi A., Bonacci S., Paolino D., Celia C., Procopio A., Fresta M., Cosco D. (2019). Paclitaxel-loaded sodium deoxycholate-stabilized zein nanoparticles: Characterization and in vitro cytotoxicity. Heliyon.

[66] Merino N., Berdejo D., Bento R., Salman H., Lanz M., Maggi F., Sánchez-Gómez S., García-Gonzalo D., Pagán R. (2019). Antimicrobial efficacy of *Thymbra capitata* (L.) Cav. essential oil loaded in self-assembled zein nanoparticles in combination with heat. Ind. Crops Prod..

[67] Zhang F., Khan M.A., Cheng H., Liang L. (2019). Co-encapsulation of α-tocopherol and resveratrol within zein nanoparticles: Impact on antioxidant activity and stability. J. Food Eng..

[68] Hu K., Huang X., Gao Y., Huang X., Xiao H., McClements D.J. (2015). Core-shell biopolymer nanoparticle delivery systems: Synthesis and characterization of curcumin fortified zein-pectin nanoparticles. Food Chem..

[69] Huang X., Dai Y., Cai J., Zhong N., Xiao H., McClements D.J., Hu K. (2017). Resveratrol encapsulation in core-shell biopolymer nanoparticles: Impact on antioxidant and anticancer activities. Food Hydrocoll..

[70] Khan M.A., Yue C., Fang Z., Hu S., Cheng H., Bakry A.M., Liang L. (2019). Alginate/chitosan-coated zein nanoparticles for the delivery of resveratrol. J. Food Eng..

[71] Sun C., Dai L., Gao Y. (2017). Interaction and formation mechanism of binary complex between zein and propylene glycol alginate. Carbohydr. Polym..

[72] Chen H., Zhong Q. (2015). A novel method of preparing stable zein nanoparticle dispersions for encapsulation of peppermint oil. Food Hydrocoll..

[73] Kibici D., Kahveci D. (2019). Effect of Emulsifier Type, Maltodextrin, and β-Cyclodextrin on Physical and Oxidative Stability of Oil-In-Water Emulsions. J. Food Sci..

[74] Schmitt V., Cattelet C., Leal-Calderon F. (2004). Coarsening of alkane-in-water emulsions stabilized by nonionic poly (oxyethylene) surfactants: The role of molecular permeation and coalescence. Langmuir.

[75] Urbina-Villalba G., Forgiarini A., Rahn K., Lozsán A. (2009). Influence of flocculation and coalescence on the evolution of the average radius of an O/W emulsion. Is a linear slope of R [combining macron] 3 vs. t an unmistakable signature of Ostwald ripening?. Phys. Chem. Chem. Phys..

[76] Guerra-Rosas M.I., Morales-Castro J., Ochoa-Martínez L.A., Salvia-Trujillo L., Martín-Belloso O. (2016). Long-term stability of food-grade nanoemulsions from high methoxyl pectin containing essential oils. Food Hydrocoll..

[77] Li X., Wang L., Wang B. (2017). Optimization of encapsulation efficiency and average particle size of Hohenbuehelia serotina polysaccharides nanoemulsions using response surface methodology. Food Chem..

[78] Zhang J., Bing L., Reineccius G.A. (2016). Comparison of modified starch and Quillaja saponins in the formation and stabilization of flavor nanoemulsions. Food Chem..

[79] Zhou X., Chen L., Han J., Shi M., Wang Y., Zhang L., Li Y., Wu W. (2017). Stability and physical properties of recombined dairy cream: Effects of soybean lecithin. Int. J. Food Prop..

[80] Patel A.R., Velikov K.P. (2014). Zein as a source of functional colloidal nano-and microstructures. Curr. Opin. Colloid Interface Sci..

[81] Chen B., Li H., Ding Y., Suo H. (2012). Formation and microstructural characterization of whey protein isolate/beet pectin coacervations by laccase catalyzed cross-linking. LWT Food Sci. Technol..

[82] Ryu V., McClements D.J., Corradini M.G., Yang J.S., McLandsborough L. (2018). Natural antimicrobial delivery systems: Formulation, antimicrobial activity, and mechanism of action of quillaja saponin-stabilized carvacrol nanoemulsions. Food Hydrocoll..

[83] Tippel J., Lehmann M., von Klitzing R., Drusch S. (2016). Interfacial properties of Quillaja saponins and its use for micellisation of lutein esters. Food Chem..

[84] Shukla R., Cheryan M. (2001). Zein: The industrial protein from corn. Ind. Crops Prod..

[85] Jones O.G., Lesmes U., Dubin P., McClements D.J. (2010). Effect of polysaccharide charge on formation and properties of biopolymer nanoparticles created by heat treatment of β-lactoglobulin–pectin complexes. Food Hydrocoll..

[86] Chen S., Han Y., Wang Y., Yang X., Sun C., Mao L., Gao Y. (2019). Zein-hyaluronic acid binary complex as a delivery vehicle of quercetagetin: Fabrication, structural characterization, physicochemical stability and in vitro release property. Food Chem..

[87] McClements D.J. (2015). Encapsulation, protection, and release of hydrophilic active components: Potential and limitations of colloidal delivery systems. Adv. Colloid Interface Sci..

[88] Ali A., Le Potier I., Huang N., Rosilio V., Cheron M., Faivre V., Turbica I., Agnely F., Mekhloufi G. (2018). Effect of high pressure homogenization on the structure and the interfacial and emulsifying properties of β-lactoglobulin. Int. J. Pharm..

[89] Ali A., Mekhloufi G., Huang N., Agnely F. (2016). β-lactoglobulin stabilized nanemulsions—Formulation and process factors affecting droplet size and nanoemulsion stability. Int. J. Pharm..

[90] Zhang Y., Niu Y., Luo Y., Ge M., Yang T., Yu L.L., Wang Q. (2014). Fabrication, characterization and antimicrobial activities of thymol-loaded zein nanoparticles stabilized by sodium caseinate-chitosan hydrochloride double layers. Food Chem..

[91] Ben Jemaa M., Falleh H., Neves M.A., Isoda H., Nakajima M., Ksouri R. (2017). Quality preservation of deliberately contaminated milk using thyme free and nanoemulsified essential oils. Food Chem..

[92] Wikiera A., Grabacka M., Byczyński Ł., Stodolak B., Mika M. (2021). Enzymatically Extracted Apple Pectin Possesses Antioxidant and Antitumor Activity. Molecules.

[93] Peña-Ramos E.A., Xiong Y.L. (2001). Antioxidative Activity of Whey Protein Hydrolysates in a Liposomal System. J. Dairy Sci..

[94] Ramadan M.F. (2008). Quercetin increases antioxidant activity of soy lecithin in a triolein model system. LWT Food Sci. Technol..

[95] Zhang B., Luo Y., Wang Q. (2011). Effect of acid and base treatments on structural, rheological, and antioxidant properties of α-zein. Food Chem..

[96] Sarabandi K., Jafari S.M., Mahoonak A.S., Mohammadi A. (2019). Application of gum Arabic and maltodextrin for encapsulation of eggplant peel extract as a natural antioxidant and color source. Int. J. Biol. Macromol..

[97] Pitalua E., Jimenez M., Vernon-Carter E.J., Beristain C.I. (2010). Antioxidative activity of microcapsules with beetroot juice using gum Arabic as wall material. Food Bioprod. Process..

[98] Karaaslan M., Şengün F., Cansu Ü., Başyiğit B., Sağlam H., Karaaslan A. (2021). Gum arabic/maltodextrin microencapsulation confers peroxidation stability and antimicrobial ability to pepper seed oil. Food Chem..

[99] Ali K.S.E., Salih T.A.A., Daffalla H.M. (2020). In vitro phytochemical, larvicidal and antimicrobial activities of gum arabic extract. Walailak J. Sci. Technol..

[100] Heim K.E., Tagliaferro A.R., Bobilya D.J. (2002). Flavonoid antioxidants: Chemistry, metabolism and structure-activity relationships. J. Nutr. Biochem..

[101] Radünz M., da Trindade M.L.M., Camargo T.M., Radünz A.L., Borges C.D., Gandra E.A., Helbig E. (2019). Antimicrobial and antioxidant activity of unencapsulated and encapsulated clove (*Syzygium aromaticum*, L.) essential oil. Food Chem..

[102] Moghimi R., Ghaderi L., Rafati H., Aliahmadi A., McClements D.J. (2016). Superior antibacterial activity of nanoemulsion of Thymus daenensis essential oil against E. coli. Food Chem..

[103] Anwer M.K., Jamil S., Ibnouf E.O., Shakeel F. (2014). Enhanced antibacterial effects of clove essential oil by nanoemulsion. J. Oleo Sci..

[104] Maté J., Periago P.M., Palop A. (2016). When nanoemulsified, d-limonene reduces Listeria monocytogenes heat resistance about one hundred times. Food Control.

[105] Zahi M.R., Liang H., Yuan Q. (2015). Improving the antimicrobial activity of D-limonene using a novel organogel-based nanoemulsion. Food Control.

[106] Xue J., Davidson P.M., Zhong Q. (2015). Antimicrobial activity of thyme oil co-nanoemulsified with sodium caseinate and lecithin. Int. J. Food Microbiol..

[107] Donsi F., Annunziata M., Vincensi M., Ferrari G. (2012). Design of nanoemulsion-based delivery systems of natural antimicrobials: Effect of the emulsifier. J. Biotechnol..

